# Multivariable analysis of the association between fathers’ and youths’ physical activity in the United States

**DOI:** 10.1186/1471-2458-13-1075

**Published:** 2013-11-14

**Authors:** Zeynep Isgor, Lisa M Powell, Youfa Wang

**Affiliations:** 1 Department of Economics and Institute for Health Research and Policy, University of Illinois at Chicago, Chicago, IL, USA; 2 Health Policy and Administration, School of Public Health and Institute for Health Research and Policy, University of Illinois at Chicago, Chicago, IL, USA; 3 Department of Social and Preventive Medicine, School of Public Health and Health Professions, University at Buffalo, State University of New York, Buffalo, NY, USA

**Keywords:** Child, Adolescent, Physical activity, Resemblance, Parent

## Abstract

**Background:**

Although the benefits of physical activity have been well-established, a significant number of children and adolescents in the U.S. do not meet the recommended levels of daily physical activity. Parental influences such as parents’ physical activity participation may play an important role in affecting youths’ physical activity.

**Methods:**

This study used the Child Development Supplement of the Panel Study of Income Dynamics to examine the associations between fathers’ vigorous physical activity (VPA) and VPA participation (>0 day(s)/week) and frequency (days/week) of 887 youths aged 10 through 18 based on a nationally representative sample of families in the US. Logistic and negative binomial regression analyses were used to examine the association between past frequency (times/week) and an indicator of recommended (≥3 times/week) frequency of father’s VPA and youth’s VPA participation and youths’ VPA frequency, respectively. We examined the sensitivity to the inclusion of various youth, family, mother’s VPA, and contextual control measures. Analyses also were undertaken by gender.

**Results:**

Father’s past VPA frequency was positively associated with both youths’ VPA participation and with youths’ VPA frequency for the full and female samples of youths, even after the inclusion of demographic, socio-economic, and local area characteristics. Father’s past recommended VPA frequency was positively associated with youths’ VPA participation in the full sample only and with youths’ VPA frequency in the female sub-sample only. Simulation results showed that an increase in father’s past weekly VPA frequency from zero to the minimum recommended level (three times per week) was associated with an increased predicted probability of youths’ weekly VPA participation from approximately 67% to 74% for the full sample (61% to 73% for females and 74% to 77% for males).

**Conclusion:**

The results from this study suggest that environmental and/or family based interventions that increase fathers’ VPA may help improve youths’ VPA.

## Background

The health benefits of a physically active life-style in adulthood are well-documented [1]. There is also evidence in support of immediate health benefits of physical activity (PA) for children and adolescents, in particular for obesity prevention [2]. Several reviews emphasized the benefits of PA even among healthy children [3-5]. Furthermore, studies have reported a moderate tracking of PA from childhood into adulthood [6,7]. Nonetheless, a significant number of American youths do not participate in sufficient levels of PA [8]. Therefore, it is important to identify the determinants of PA among youths.

Social and environmental factors have frequently been the foci of research aimed at identifying the determinants of youth PA behavior. Among the social factors, research on the associations between parenting practices/styles and/or behaviors and youth PA participation constituted an important area of investigation. Such research often founded its framework in “Social Cognitive Theory” [9], which emphasizes that the formation of behavior entails learning by observing with related hypotheses that children/adolescents model their behavior after those agents to whom their exposure (i.e. opportunity of observation) is greatest and/or for whom they nourish the most respect, love, and admiration. This implies that parental PA behavior may exert influences on youths’ PA behavior.

Several review studies that summarized the findings of research on the relationship between parental and youth PA have been published. A recent review by Trost and Loprinzi [10] (2011) concluded that there was no consistent evidence of an association between parental and youth PA or, at best, such evidence was weak. Another recent review by Edwardson and Gorely [11] (2010) found no association between maternal self-reported PA and adolescents’ vigorous physical activity (VPA), but reported that paternal self-reported VPA was positively associated with the adolescents’ VPA.

A meta-analysis of various types of parental behavior conducted by Pugliese and Tinsley [12] (2007) indicated that parents’ own PA levels, referred to as “modeling behavior of parents”, exhibited the weakest relation in regards to youths’ PA among all the parental behaviors that were positively and significantly associated with youths’ PA. Gustafson and Rhodes [13] (2006) reported mixed evidence regarding the parental modeling of PA behavior on youth PA. Examining the relationship between parental modeling of PA and children’s PA, a review study by Ferreira et al. [14]. (2006) concluded that no relevant associations were found in those studies that did not separate mother’s PA from that of father’s, whereas among those studies that did so, father’s PA levels were reported to be positively and significantly related to children’s PA levels in 52% of the cases and mother’s PA levels were mostly found to be unrelated. As for adolescents, the same review study concluded that although modeling of PA from parents did not exhibit any significant associations, this lack of association was not necessarily conclusive in regards to father’s PA levels since father’s PA levels were observed in less than 60% of the cases. Sallis et al. [15]. (2000) reported that less than 40% of the studies covering the topic reported a significant relationship between parental and child activity.

Only a limited number of previous studies contained information on paternal PA levels and the ones that were able to examine paternal and maternal PA levels separately in relation to youth PA reported that paternal compared to maternal PA was more often associated with youth PA. Consequently, this study built on the existing literature by further examining associations between fathers’ self-reported weekly VPA levels and their children’s VPA outside of school among a sample of youths aged 10 through 18 drawn from a nationally representative sample of families in the U.S, while also controlling for mothers’ VPA levels and important physical activity-related contextual factors. Furthermore, for the full sample of youths, as well as by gender, we assessed the robustness of the association between father’s and youth’s VPA to alternative model specifications. We used past (lagged t-2) father’s and mother’s VPA to address potential endogeneity that may arise from reverse causality.

## Methods

### Data

The Child Development Supplement (CDS) of the Panel Study of Income Dynamics (PSID) data were collected by the University of Michigan’s Institute for Social Research (ISR) as a supplement to focus on children of the PSID sample. PSID is a nationally representative longitudinal data set collected since 1968 providing rich information with a focus on income and human capital measures. In 1997, the PSID supplemented its core data collection with additional information on PSID parents and their 0–12 year-old children (CDS-I). For the second wave of CDS in 2002/2003 (CDS-II), 2017 families (91%) in CDS-I were successfully re-interviewed, providing information on 2907 children aged 5–18 years. This study used the CDS-II data collected in 2002/2003, since the PA outcome measures were only available in the second wave (CDS-II) for youths who were 10 years of age or older. The final sample based on non-missing data that we used in our analyses included 887 youths aged 10 through 18 all of whom resided in two-parent families over a period of two years (2001 through 2003) and for whom both father’s and mother’s PA were reported in the 2001 and 2003 PSID family interviews.

### Outcome, key exposure, and control measures

Our two outcome measures for youth PA participation were based on the question: “Including everything you do outside of a physical education (PE) class, how many days a week do you get at least 30 minutes of VPA?” Our first outcome measure, youths’ VPA participation, was a dichotomous indicator generated based on whether the adolescent participated in VPA outside of a PE class for at least 30 minutes on at least one day a week. The second outcome variable, youths’ VPA frequency, was a continuous measure of the number of days per week the youth engaged in at least 30 minutes of VPA outside of a PE class.

Our first key exposure measure of father’s past (lagged t-2 = 2001) VPA frequency and the analogous control measure of mother’s past (lagged t-2 = 2001) VPA frequency were based on the following question asked, with two prompts one for inquiring number of times of VPA episodes and one for inquiring the time unit for the number mentioned in the first prompt, to the family heads for themselves and for their wives in the 2001 PSID survey: “How often do you/does your wife participate in VPA or sports---such as heavy housework, aerobics, running, swimming, or bicycling?” and were constructed as continuous measures of weekly number of times of father’s and mother’s VPA. Self-reported values greater than or equal to 14 times a week were top- coded as 14 to eliminate potential outliers. Our second key exposure measure and the analogous control measure that represented father’s and mother’s past recommended VPA frequency, respectively, were defined as parent’s past weekly VPA participation greater than equal to 3, in order to approximate the recent adult aerobic physical activity guidelines for important health benefits of the Centers for Disease Control and Prevention (CDC), which recommended 1 hour and 15 minutes (75 minutes) of vigorous-intensity aerobic activity (i.e., jogging or running) every week [16].

Individual and family-level demographic and socio-economic control measures included age, gender, race/ethnicity (white, black, Hispanic, other race); family income (quintiles representing low, near-low, middle, near-high, and high), mother’s past VPA frequency (number of times per week) or mother’s past recommended VPA frequency (≥3 times/week), maternal education (less than high school, high school, some college, college or more, missing), and maternal work hours (not work, work part-time, work full-time). Information on the family background variables was drawn from the 2003 PSID survey and linked to the CDS data by family identifiers. Information on mother’s past VPA frequency and mother’s past recommended VPA frequency was drawn from the 2001 PSID survey and linked to the CDS data by family identifiers.

Contextual control measures included the zip code-level degree of urbanization (urban, suburban, or rural/farm), median household income, and commercial PA-related facility availability. The degree of urbanization and the median household income measures were drawn from Census 2000 [17,18] and matched to the CDS by zip code. A zip code’s degree of urbanization was represented by the dichotomous measure indicating the category (urban, suburban, or rural/farm) making up the largest percentage of its population. The commercial PA-related facility outlet data were obtained from a business list developed by Dun and Bradstreet [19] (D&B) available through MarketPlace software for the year 2003 and were based on 16 separate 8-digit SIC codes that included dance schools; sports instruction schools, camps and services; and, specific sports instruction schools for baseball, basketball, swimming, physical fitness, marital arts, yoga, gymnastic, hockey, and ice/roller skating. These facility outlet data were matched to the CDS by year and zip code, and computed as the number of available outlets per 10,000 capita per 10 square miles using Census 2000 population and land area estimates.

### Analyses

We examined the association between fathers’ past VPA frequency (i.e. times/week) and recommended VPA frequency (≥3 times/week) and youths’ VPA participation (i.e. >0 day(s)/week) and VPA frequency (days/week) outside of school PE classes. Multi-variable regression analyses that controlled for individual, family, and contextual factors were used to test our hypotheses of whether higher frequency of fathers’ past VPA or whether father’s past recommended VPA frequency were positively associated with (1) the probability of youths’ VPA participation and (2) the youths’ VPA frequency, for at least 30 minutes a day outside the school PE classes.

The empirical fully-adjusted model was represented by Equation (1) and the partially-adjusted models were represented by Equations (2) through (4) below:

(1) $$\begin{array}{l} PA_{\mathrm{ifs}}=\beta_{10}+\beta_{11}\mathrm{FP}A_{f}+\beta_{12}X_{i}+\beta_{13}\mathrm{MP}A_{f}\\ \;+{\beta_{1}}_{4}Z_{f}+\beta_{15}N_{s}+\epsilon_{\mathrm{ifs}} \end{array}$$

(2) $$PA_{\mathrm{ifs}}=\beta_{20}+\beta_{21}\mathrm{FP}A_{f}+\beta_{22}X_{i}+\beta_{23}Z_{f}+\beta_{24}N_{s}+\epsilon_{\mathrm{ifs}}$$

(3) $${\mathrm{PA}}_{\mathrm{ifs}}=\beta_{30}+\beta_{31}{\mathrm{FPA}}_{f}+\beta_{32}X_{i}+\beta_{33}Z_{f}+\epsilon_{\mathrm{ifs}}$$

(4) $$PA_{\mathrm{ifs}}=\beta_{40}+\beta_{41}\mathrm{FP}A_{f}+\beta_{42}X_{i}+\epsilon_{\mathrm{ifs}}$$

In these equations, PA_ifs_ represented the two outcome measures defined above pertaining to youth VPA. FPA_f_ represented either one of the two key exposure measures of father’s past VPA frequency or father’s past recommended VPA. *X*_*i*_ was a vector of youth characteristics for youth *i* and included age, gender, and race. MPA_f_ was a control measure representing the mother’s past VPA frequency or past recommended VPA frequency. *Z*_*f*_ was a vector of family characteristics for family *f* and included family income, mother’s education level, and mother’s work hours. *N*_*s*_ was a vector of contextual measures for each zip code *s*, referred to as a given youth’s neighborhood/local area, and included degree of urbanization, a continuous measure of median household income, and a continuous measure of commercial PA-related facility availability.

We estimated logistic regression models for the probability of youths’ weekly VPA participation and reported the odds ratios (i.e. exponentiated coefficients) and 95% confidence intervals. For our continuous outcome measure of youths’ VPA frequency, we estimated a negative binomial count regression model from which incident rate ratios (i.e. exponentiated coefficients) and 95% confidence interval estimates were reported. We estimated a negative binomial count regression model rather than a Poisson count regression model since the over-dispersion parameter was statistically significant indicating that the data were over-dispersed. For all our regression estimates, robust standard errors that were clustered at the family level were used.

The fully-adjusted model (Equation 1) included all youth, maternal and family, and contextual control measures. The first partially-adjusted model (Equation 2) included all control measures with the exception of mother’s past VPA. The second partially-adjusted model (Equation 3) included all control measures with the exceptions of mother’s past VPA and contextual factors. The third partially-adjusted model (Equation 4) included only youth (i.e. age, gender, race) characteristics as control measures. We estimated all four models for both outcome measures and for both key exposure measures for the full sample of youths as well as separately for the male and female sub-samples. We performed all analyses using STATA version 12.0, College Station, Texas. All regression analyses performed were weighted using the appropriate CDS sample weights [20]. This study was approved by the Institutional Review Board of the University of Illinois at Chicago.

## Results

Descriptive statistics are reported in Table 1 for the full (N = 887), male (N = 453), and female (N = 434) samples and were weighted. The prevalence of weekly VPA participation among youths was 67.83% (72.02% for males and 63.58% for females). Youths participated in VPA, on average, 3.05 days per week (3.34 days/week for males and 2.75 days/week for females). The median frequency of youth VPA was 3 days per week (not reported in Table 1). Fathers’ past VPA frequency and past recommended frequent VPA participation was 2.02 times/week and 29.94% on average, respectively, for the full sample. The corresponding numbers for mothers’ past VPA were 2.00 times/week and 34.08%.

**Table 1:** Descriptive statistics: outcome measures, key exposure measures, and control measures

	**Mean (SD) / Frequency**		
	**Full sample**	**Male sample**	**Female sample**
Outcome measures:			
Youth participates in any weekly vigorous physical activity (VPA)	67.83%	72.02%	63.58%
Number of days per week youth participates in VPA	3.05 (2.59)	3.34 (2.59)	2.75 (2.56)
Key exposure measures:			
Father’s lagged (t-2) weekly number of times of VPA	2.02 (2.67)	1.87 (2.50)	2.17 (2.84)
Father meets recommended (≥3 times/week) VPA	29.94%	27.66%	32.25%
Control measures:			
Child’s age	13.83 (2.51)	13.65 (2.52)	14.00 (2.48)
Child’s gender is male	50.29%		
Child’s race & ethnicity			
White*	72.34%	72.06%	72.63%
African-American	8.12%	10.89%	5.32%
Hispanic	13.54%	11.03%	16.08%
Other race	5.99%	6.01%	5.96%
Mother’s lagged (t-2) weekly number of times VPA	2.00 (2.36)	2.13 (2.50)	1.86 (2.20)
Mother meets recommended (≥3 times/week) VPA	34.08%	36.86%	31.27%
Mother’s education			
Less than high school*	13.05%	12.65%	13.46%
High school graduate	27.43%	24.45%	30.44%
Completed some college	26.93%	29.97%	23.85%
Completed college or more	26.93%	28.48%	25.36%
Missing	5.66%	4.45%	6.89%
Mother’s work status			
Does not work*	18.24%	18.51%	17.96%
Works part-time	39.69%	34.25%	42.16%
Works full-time	42.07%	44.25%	39.88%
Family income (in year 2003 dollars) ('000)	92.39 (120.63)	94.35 (128.71)	90.40 (111.99)
Local area physical activity-related facility availability	1.02 (2.43)	1.14 (2.85)	0.89 (1.92)
Local area median household income (in year 2000 dollars) ('000)	47.64 (18.98)	46.80 (18.72)	48.49 (19.21)
Local area degree of urbanization			
Urban*	66.86%	67.78%	65.93%
Suburban	12.42%	10.99%	13.86%
Rural/Farm	20.73%	21.24%	20.21%
N	887	453	434

Notes: Summary Statistics are weighted. Standard deviations (SD) are shown in parenthesis for continuous variables. * Denotes reference categories in regression models. (t-2) refers to two-year lagged values of father’s and mother’s VPA. Physical activity-related facility availability variable is defined per 10,000 capita per 10 square miles. Median household income measure is obtained from the Census 2000 Data and is at the zip code level.

The results for the logistic regression analyses presented in Panel 1 of Table 2 show that, based on the fully-adjusted model (Equation 1), a one unit (i.e. times/week) increase in father’s past VPA frequency was associated with a 13.4% increase in the odds of youths weekly VPA participation (18.8% for females and 5.3% for males, but this association was not statistically significant for males), holding all other variables constant.

**Table 2:** **Association between father’s past vigorous physical activity**^**a **^**(VPA) frequency and youth’s VPA**

	**Panel 1: Whether youth participates in weekly VPA**			
	**Odds ratio (95% confidence interval)**			
	**Model 1**	**Model 2**	**Model 3**	**Model 4**
All (N = 887)	1.134***	1.141***	1.135***	1.145***
	(1.042 - 1.234)	(1.046 - 1.243)	(1.043 - 1.236)	(1.049 - 1.250)
Male (N = 453)	1.053	1.060	1.060	1.057
	(0.940 - 1.180)	(0.943 - 1.191)	(0.944 - 1.191)	(0.941 - 1.189)
Female (N = 434)	1.188***	1.186***	1.171***	1.200***
	(1.059 - 1.332)	(1.057 - 1.331)	(1.048 - 1.308)	(1.069 - 1.347)
	**Panel 2: Youths’ weekly VPA frequency (i.e. days/week)**			
	**Incident rate ratio (95% confidence interval)**			
	**Model 1**	**Model 2**	**Model 3**	**Model 4**
All (N = 887)	1.026**	1.028**	1.027**	1.031**
	(1.001 - 1.052)	(1.003 - 1.054)	(1.003 - 1.053)	(1.006 - 1.057)
Male (N = 453)	0.995	0.996	0.997	0.996
	(0.959 - 1.032)	(0.960 - 1.034)	(0.961 - 1.034)	(0.961 - 1.031)
Female (N = 434)	1.047***	1.051***	1.045***	1.055***
	(1.014 - 1.082)	(1.017 - 1.086)	(1.013 - 1.079)	(1.022 - 1.089)
Control measures:				
Child characteristics^b^	YES	YES	YES	YES
Family characteristics^c^	YES	YES	YES	NO
Contextual measures^d^	YES	YES	NO	NO
Mother’s past VPA^e^	YES	NO	NO	NO

Notes: Odd ratios (estimated coefficients “β_i_” transformed to “e^βi^”) and exponentiated confidence intervals in parenthesis that are robust and clustered using family identifiers are reported from the logistic proability regression analysis for the “Whether Youth Participates in Any Weekly VPA” outcome.

Rate ratios (estimated coefficients “β_i_” transformed to “e^βi”^ ) and exponentiated confidence intervals in parenthesis that are robust and clustered using family identifiers are reported from negative binomial count data regression analysis for the “Number of Days per Week Youth Participates in VPA” outcome.

All regressions are weighted.

*** denotes significance at p≤0.01; ** denotes significance at p≤0.05; + denotes significance at p≤0.10.

^a^ The independent variable of interest, “Father’s past vigorous physical activity frequency” is a continuous measure of weekly number of times of father’s past (lagged t-2 = 2001) VPA.

^b^ Child characteristics include the following measures: Age, gender, race.

^c^Family Characteristics include the following measures: Mother’s Education, Mother’s Work Hours, Family Income.

^d^Contextual Measures include the following: Local Area Physical Activity-Related Facility Availability, Local Area Median Household Income, Local Area Degree of Urbanization.

^e^ “Mother’s Past VPA” is the lagged (t-2 = 2001) weekly number of times of vigorous physical activity participation of child’s mother.

McFadden’s Adjusted R-squares for Model 1 (i.e. fully-adjusted model) from the logistic regression analyses reported in Panel 1 are 0.173, 0.137, and 0.258 for the full, male, and female sub-samples, respectively.

McFadden’s Adjusted R-squares for Model 1 (i.e. fully-adjusted model) from the negative binomial regression analyses reported in Panel 2 are 0.026, 0.015, and 0.044 for the full, male, and female sub-samples, respectively.

The odds ratio estimates reported in Panel 1 of Table 2 for the analysis of youths’ weekly VPA participation outcome did not vary much across the four models. Accordingly, for a unit change in father’s past VPA frequency, the odds of youths’ weekly VPA participation increased by a factor of 1.13 in the fully-adjusted model (Equation 1) to 1.15 in the most parsimonious partially-adjusted model (Equation 4), holding all other variables constant, and were statistically significant (p ≤ 0.05) in all models for the full sample of youths. Similar patterns regarding the magnitudes of estimates between the fully- and partially-adjusted models also were found for the male and female subsamples.

The results from the count model presented in Panel 2 of Table 2 show that a one unit increase in father’s past VPA frequency was significantly associated with a 2.6% and a 4.7% higher frequency of youths’ weekly VPA for the full and female samples, respectively, in the fully-adjusted model. This association was close to zero and not found to be statistically significant for the male sample. Across models (ranging from the fully-adjusted model to the most parsimonious partially-adjusted model), the magnitudes of the estimates for the analysis of youths’ weekly VPA frequency ranged from 2.6% to 3.1% for the full sample and from 4.7% to 5.5% for the female sample.

Table 3 reports the findings related to youths’ exposure to fathers’ past recommended frequent VPA. As shown in Panel 1, for the full sample for the fully-adjusted model, the odds of any weekly VPA participation for those youths whose fathers’ past VPA frequency was at or above recommended levels was 86.2% higher than the odds of any weekly VPA participation for those youths whose fathers’ past VPA frequency was below recommended levels. The corresponding results by gender (a higher odds of weekly VPA of 87.2% for females and 53.7% for males) were only weakly statistically significant for females. As seen in Panel 1 of Table 3, the magnitudes of odds ratio estimates ranged from 1.86 in the fully-adjusted model (Equation 1) to 2.05 in the most parsimonious partially-adjusted model (Equation 4) and all estimates were statistically significant for the full sample of youths. By gender, the magnitudes of odds ratio estimates did not vary much across the four models, were not statistically significant for the male sub-sample, and were statistically significant (i.e. p ≤ 0.05) for the female sub-sample only in the partially-adjusted models, but not in the fully-adjusted model.

**Table 3:** **Association between father’s past recommended vigorous physical activity**^**a **^**(VPA) frequency and youth’s VPA**

	**Panel 1: Whether youth participates in weekly VPA**			
	**Odds ratio(95% confidence interval)**			
	**Model 1**	**Model 2**	**Model 3**	**Model 4**
All (N = 887)	1.862***	1.928***	1.921***	2.054***
	(1.195 - 2.902)	(1.234 - 3.11)	(1.228 - 3.004)	(1.300 - 3.246)
Male (N = 453)	1.537	1.567	1.589	1.590
	(0.835 - 2.830)	(0.849 - 2.893)	(0.859 - 2.939)	(0.855 - 2.959)
Female (N = 434)	1.872^+^	1.964**	1.920**	2.368***
	(0.976 - 3.592)	(1.026 - 3.762)	(1.012 - 3.642)	(1.251 - 4.483)
	**Panel 2: Youths’ weekly VPA frequency (i.e. days/week)**			
	**Incident rate ratio (95% confidence interval)**			
	**Model 1**	**Model 2**	**Model 3**	**Model 4**
All (N = 887)	1.140^+^	1.154^+^	1.156^+^	1.184**
	(0.980 - 1.327)	(0.993 - 1.342)	(0.994 - 1.345)	(1.015 - 1.382)
Male (N = 453)	0.987	0.997	0.996	0.983
	(0.805 - 1.211)	(0.814 - 1.221)	(0.813 - 1.221)	(0.807 - 1.198)
Female (N = 434)	1.310**	1.322**	1.309**	1.427***
	(1.044 - 1.644)	(1.053 - 1.660)	(1.045 - 1.641)	(1.140 - 1.786)
Control measures:				
Child characteristics^b^	YES	YES	YES	YES
Family characteristics^c^	YES	YES	YES	NO
Contextual measures^d^	YES	YES	NO	NO
Mother’s past recommended VPA^e^	YES	NO	NO	NO

Notes: Odd ratios (estimated coefficients “β_i_” transformed to “e^βi^”) and exponentiated confidence intervals in parenthesis that are robust and clustered using family identifiers are reported from the logistic proability regression analysis for the “Whether Youth Participates in Any Weekly VPA” outcome.

Rate ratios (estimated coefficients “β_i_” transformed to “e^βi”^ ) and exponentiated confidence intervals in parenthesis that are robust and clustered using family identifiers are reported from negative binomial count data regression analysis for the “Number of Days per Week Youth Participates in VPA” outcome.

All regressions are weighted.

*** denotes significance at p≤0.01; ** denotes significance at p≤0.05; + denotes significance at p≤0.10.

^a^ The independent variable of interest, “Father’s Past Recommended Vigorous Physical Activity Frequency”, is a binary indicator of lagged (t-2 = 2001) recommended weekly number of times of VPA, which takes a value of “1” if father’s past (t-2 = 2001) recommended VPA is ≥3, and “0” otherwise.

^b^Child characteristics include the following measures: Age, gender, race

^c^Family Characteristics include the following measures: Mother’s Education, Mother’s Work Hours, Family Income

^d^Contextual Measures include the following: Local Area Physical Activity-Related Facility Availability, Local Area Median Household Income, Local Area Degree of Urbanization

^e^ “Mother’s Past Recommended VPA” is the lagged (t-2 = 2001) recommended (≥3 times/week) weekly number of times of VPA participation of child’s mother.

McFadden’s Adjusted R-squares for Model 1 (i.e. fully-adjusted model) from the logistic regression analyses reported in Panel 1 are 0.169, 0.137, and 0.247 for the full, male, and female sub-samples, respectively.

McFadden’s Adjusted R-squares for Model 1 (i.e. fully-adjusted model) from the negative binomial regression analyses reported in Panel 2 are 0.025, 0.015, and 0.043 for the full, male, and female sub-samples, respectively.

As shown in Panel 2 of Table 3, fathers’ past recommended VPA frequency was significantly associated with a 31% increase in youths’ weekly VPA frequency in the fully-adjusted model for the females. As for the full sample, the magnitude of the associations did not vary much across the four models and was found to be statistically significant (p ≤ 0.05) for the model that included only the child characteristics as control measures (Equation 4). By gender, the associations were around zero and not significant in the fully- or in any of the partially-adjusted models for the male sub-sample of youths, whereas the magnitude of associations ranged from 1.31 in the fully-adjusted model to 1.43 in the most parsimonious partially-adjusted model and were statistically significant in all models for the female sub-sample of youths.

Mother’s past VPA frequency and mother’s past recommended VPA frequency were controlled in the analyses using the first key exposure measure of father’s past VPA frequency and the second key exposure measure of father’s past recommended VPA frequency, respectively. Neither one of the mother’s past VPA measures was found to be significantly associated with any one of the youth’s VPA outcomes.

Finally, to help further interpret the implications of our results, we undertook a series of simulations for the full, male and female samples. Based on our regression estimates from the fully-adjusted models, we estimated the predicted probability and frequency of youths’ weekly VPA participation for four different simulation scenarios: 1) using the original data values of the predictor variables; 2) increasing father’s weekly VPA frequency by one standard deviation; 3) setting father’s weekly VPA frequency to zero times per week; and, 4) setting father’s weekly VPA frequency to the minimum recommended level of three times per week for all observations.

As reported in Panel 1 of Table 4, evaluated at the original data values of the variables, our model predicted youths’ weekly VPA participation to be approximately 72%, 76%, and 69%, respectively, for the full, male, and female samples of youths. Our simulation results from scenario 2 revealed that a one standard deviation increase in father’s past weekly VPA frequency yielded a predicted probability in the range of 78-79% in youths’ weekly VPA participation for the full, female, and male samples. Furthermore, our simulation results from scenarios 3 and 4 demonstrated that an increase in father’s past weekly VPA frequency from zero to the minimum recommended level (three times per week) was associated with an increased predicted probability of youths’ weekly VPA participation from approximately 67% to 74% for the full sample, 61% to 73% for female youths, and 74% to 77% for male youths.

**Table 4:** Simulation analyses of changes in father’s past weekly VPA frequency on youths’ weekly VPA participation and frequency

	**Panel 1: Logistic regression of fully-adjusted model**		
	**Predicted probability of youths’ weekly VPA participation (%)**		
Simulation scenarios:	Full sample	Male sample	Female sample
Baseline (i.e. no change) in father’s past weekly VPA frequency	72.05	76.26	69.27
A one SD increase in father’s past weekly VPA frequency^a^	78.30	78.52	78.60
Father’s past weekly VPA frequency set to 3.0^b^	74.47	77.30	72.92
Father’s past weekly VPA frequency set to zero	66.67	74.46	60.83
	**Panel 2: Negative binomial regression of fully-adjusted model**		
	**Predicted rate of youths’ weekly VPA frequency (days/week)**		
Simulation scenarios:	Full sample	Male sample	Female sample
Baseline (i.e. no change) in father’s past weekly VPA frequency	2.85	3.22	2.42
A one SD increase in father’s past weekly VPA frequency^a^	3.06	3.18	2.76
Father’s past weekly VPA frequency set to 3.0^b^	2.93	3.20	2.52
Father’s past weekly VPA frequency set to zero	2.71	3.25	2.19

Notes: All simulation results are based on the fully-adjusted model. Fully-adjusted model (i.e. Equation 1) includes child and family characteristics, contextual measures, and mother’s past VPA, which are the following measures: Age, gender, race, mother’s education, mother’s work hours, family income, local area physical activity-related facility availability, local area median household income, local area degree of urbanization, mother’s past ( lagged t-2 = 2001) weekly number of times of vigorous physical activity participation.

The independent variable of interest, “Father’s past weekly vigorous physical activity frequency” is a continuous measure of weekly number of times of father’s past (lagged t-2 = 2001) VPA.

^a^ A one standard deviation (SD) increase in father’s weekly VPA frequency (i.e. times/week) is an increase of 2.67, 2.50, and 2.84 times per week, respectively, for the full, male, and female samples. These standard deviation numbers are obtained from weighted summary statistics and are shown in Table 1.

^b^ Father’s weekly VPA frequency was set to three to approximate the minimum recommendation of the recent adult aerobic physical activity guidelines for important health benefits of the Centers for Disease Control and Prevention (CDC), which recommend 1 hour and 15 minutes of vigorous-intensity aerobic activity every week, as noted in the text.

Panel 2 of Table 4 shows our simulation results from the negative binomial regressions under the same four scenarios. Evaluated at the original data values of the variables, our model predicted youths’ weekly VPA frequency to be 2.85, 3.22, and 2.42 days per week for the full, male, and female samples of youths, respectively. Under scenario 2, a one standard deviation increase in father’s past weekly VPA frequency resulted in predicted youths’ weekly VPA frequencies of 3.06, 3.18, and 2.76 days for the full, male, and female samples of youths, respectively. Our model predicted an increase in female youths’ weekly VPA frequency from 2.19 to 2.52 days and a slight reduction in male youths’ weekly VPA frequency from 3.25 to 3.20 days, as a result of an increase in father’s past weekly VPA frequency from zero to three times per week.

## Discussion

This study provided new evidence on the association between father’s and youth’s VPA frequency by 1) using a sample of youths in the U.S. derived from a nationally representative sample of families, 2) controlling for the effects of mother’s VPA behavior, 3) taking advantage of the panel nature of the PSID by studying the relationship between fathers’ past VPA and youths’ VPA that may potentially address endogeneity bias that may arise from reverse causality, 4) assessing the differential impact, if any, of father’s each additional episode of VPA participation versus father’s recommended frequent past VPA participation, 5) including a rich set of youth and family demographic and socio-economic, and contextual control measures to account for potential confounders, and 6) examining two youth PA outcomes to detect, if any, the differential impact of fathers’ past VPA on youths’ VPA participation and youths’ VPA frequency.

Our results indicated that fathers’ past weekly VPA frequency was positively and consistently associated with youths’ VPA participation and youths’ VPA frequency even after the inclusion of our complete set of control measures, including mothers’ past weekly VPA frequency, for the full and female samples of youths. We also found that fathers’ past recommended frequent VPA was positively and consistently associated with youths’ VPA participation for the full sample of youths and with youths’ VPA frequency for the female sample of youths. Furthermore, results obtained from the simulation analyses demonstrated that increases in father’s past weekly VPA frequency could improve youths’ VPA. For example, the simulation results demonstrated that an increase in father’s past weekly VPA frequency from zero to the minimum recommended level (three times per week) was associated with an increased predicted probability of youths’ weekly VPA participation from approximately 67% to 74% (from 61% to 73% for female youths and 74% to 77% for male youths). The simulation analyses results revealed the higher benefit from increases in father’s weekly VPA frequency for female youths, as compared to male youths, particularly with respect to youths’ frequency of VPA.

The results from the present study are consistent with the findings from a number of previous studies. In a study of familial correlates of adolescent girls’ PA, certain other health-related behaviors, and body composition, Bauer et al. [21]. (2011) reported that parental moderate-to-vigorous physical activity (MVPA) was positively and significantly associated with girls’ MVPA in mutually-adjusted models. However, the data used in that study included PA participation information of only one of the parents’ of each participant girl, and thus, the study was not able to examine the robustness of the association to the inclusion of the other parent’s PA, whereas in our study we were able to include maternal and paternal VPA separately while controlling for a rich set of potential confounders at the child, family, and local area levels and using a sample of children selected from a nationally representative sample of families. Crawford et al. [22]. (2010) showed that father’s role modeling of MVPA for Australian children was associated with female children’s MVPA five years later, whereas mother’s role modeling of MVPA was positively associated with the male children’s MVPA five years later. In an earlier study, DiLorenzo et al. [23]. (1998) reported that father’s PA level was significantly related to and accounted for 13% of the variance in fifth and sixth grade children’s PA. In a longitudinal study by Bauer et al. [24]. (2008), father’s, but not mother’s, attitude/care about staying fit when adolescents were in middle/high school was found to be positively and significantly associated with the weekly hours of MVPA of male adolescents five years later. Our results support the findings of these previous studies that paternal, rather than maternal, PA is associated with youths’ PA.

In contrast to our findings regarding mother’s VPA, Fuemmeler et al. [25]. (2011), in a linear regression analysis of a sample of boys and girls, reported that both the father’s and the mother’s minutes of MVPA were associated with children’s MVPA during the weekend. Findings from a longitudinal study by Cleland et al. [26]. (2011) showed that baseline maternal, but not paternal, physical activity was associated with higher levels of physical activity over time for male, but not female, elementary school children in Melbourne, Australia. Trost et al. [27]. (2003) and Loprinzi and Trost [28] (2010), using samples of US and Australian children, respectively, showed that parental PA was not directly associated with children’s PA, but an indirect association was found through parental support.

This present study is subject to certain limitations. First, the data consisted of only one cross-section for the children, making it difficult to control for potential sources of endogeneity and draw any causal conclusions. However, we did have longitudinal data for parents, which permitted us to examine the association of lagged parental PA two years back, which may have helped address potential endogeneity bias due to reverse causality. Second, the fact that the VPA participation measures were self-reported by survey participants and that the control measure of maternal VPA was reported by fathers may have caused biases. Third, due to data limitations, we were unable to address the potentially differential impact of father’s physical activity versus inactivity and/or various intensities of father’s physical activity (i.e. MVPA vs. VPA) on the youth’s PA versus inactivity behavior. Fourth, due to sample size limitations, we were unable to examine the relationship between paternal and youth PA by age group for each gender. Lastly, youths’ “vigorous” PA was not specifically defined in the CDS questionnaire, by the use of certain number of METs (metabolic equivalents) or examples of activity/sports types.

## Conclusion

American youths, especially female youths, whose fathers engage in more frequent VPA are more likely to engage in weekly VPA and also to do so more often (i.e. higher number of days/week), as compared to those youths whose fathers engage in VPA less frequently. In addition, our results suggest that youths, especially female youths, whose fathers attain recommended weekly VPA frequency are more likely to engage in weekly VPA and also to attain a higher weekly VPA frequency (i.e. days/week). These results may constitute evidence for the importance of environmental and/or family based interventions that involve increasing fathers’ VPA participation in improving youths’, especially female youths’, VPA. Future studies that are able to examine this relationship using longitudinal nationally representative data to address potential sources of biases due to time invariant unobserved heterogeneity, conducting analyses using samples of one-parent vs. two parent homes, using various types and intensities of objectively-measured PA while addressing with whom PA takes place, and examining sedentary behavior of both parents and their youths will make further contributions to the existing literature.

## Abbreviations

CDS: Child development supplement; PSID: Panel study of income dynamics; ISR: Institute for social research; D & B: Dun and Bradstreet; MVPA: Moderate-to-vigorous physical activity; VPA: Vigorous physical activity; PA: Physical activity; PE: Physical education; MET: Metabolic equivalent of task.

## Competing interests

The authors declare that they have no competing interests.

## Authors’ contributions

ZI, LMP, and YW contributed to the study design. ZI and LMP conducted the study analysis. ZI drafted the manuscript. All authors revised and approved the final manuscript.

## Pre-publication history

The pre-publication history for this paper can be accessed here:

http://www.biomedcentral.com/1471-2458/13/1075/prepub
